# Respiratory-gated micro-computed tomography imaging to measure radiation-induced lung injuries in mice following ultra-high dose-rate and conventional dose-rate radiation therapy

**DOI:** 10.1117/1.JMI.12.1.014002

**Published:** 2025-01-30

**Authors:** Nancy Lee Ford, Xi Ren, Luca Egoriti, Nolan Esplen, Stephanie Radel, Brandon Humphries, Hui-Wen Koay, Thomas Planche, Cornelia Hoehr, Alexander Gottberg, Magdalena Bazalova-Carter

**Affiliations:** a The University of British Columbia, Department of Oral Biological and Medical Sciences, Vancouver, British Columbia, Canada; b The University of British Columbia, Department of Physics and Astronomy, Vancouver, British Columbia, Canada; c The University of British Columbia, Department of Chemistry, Vancouver, British Columbia, Canada; d University of Victoria, Department of Physics and Astronomy, Victoria, British Columbia, Canada; e TRIUMF, Vancouver, British Columbia, Canada

**Keywords:** radiation-induced lung injury, respiratory-gated, micro-computed tomography imaging, radiation therapy, ultra-high dose-rate

## Abstract

**Purpose:**

Ultra-high dose-rate radiotherapy (FLASH-RT) shows the potential to eliminate tumors while sparing healthy tissues. To investigate radiation-induced lung damage, we used *in vivo* respiratory-gated micro-computed tomography (micro-CT) to monitor mice that received photon FLASH-RT or conventional RT on the FLASH irradiation research station at TRIUMF.

**Approach:**

Thirty healthy male C57BL/6 mice received baseline micro-CT scans followed by radiation therapy targeting the thorax. Treatments administered included no irradiation, 10-MV photon FLASH-RT, and 10-MV conventional RT with either 15 or 30 Gy prescribed dose. Follow-up micro-CT scans were obtained up to 24 weeks post-irradiation, and histology was obtained at the experimental endpoint. Lung volume and CT number were measured during peak inspiration and end-expiration and used to calculate the functional residual capacity (FRC) and tidal volume ($V_{T}$).

**Results:**

Radiation pneumonitis was observed sporadically in micro-CT images at 9 and 12 weeks post-irradiation. Fibrosis was observed in the endpoint images and confirmed with histology. Compared with the 15-Gy treatment groups and unirradiated controls, the micro-CT images for 30-Gy FLASH-RT showed differences during peak inspiration, with a significant reduction in $V_{T}$, whereas the 30-Gy conventional RT showed differences during end-expiration, with a significant difference in FRC from 15 Gy. Between 12 weeks and the endpoint, the 30-Gy conventional RT group exhibited the largest reduction in lung volume.

**Conclusions:**

Respiratory-gated micro-CT imaging was sensitive to radiation pneumonitis and fibrosis. Significant differences were seen in functional metrics measured at the endpoint for FRC (both 30-Gy groups) and $V_{T}$ (30-Gy FLASH-RT) compared with the control.

## 1 Introduction

Lung cancer is the leading cause of cancer death in Canada, with 21,000 deaths in 2021,^1^ with 5-year survival dropping to only 4% for stage 4 diagnoses. Treatment with thoracic radiation therapy (RT) may cause pulmonary side effects such as radiation-induced pneumonitis and fibrosis^2^ in the healthy tissue.

Ultra-high dose-rate radiotherapy (FLASH-RT) is a novel radiation treatment technology utilizing ultra-high dose-rates (UHDRs) typically exceeding 40  Gy/s to deliver treatment fractions within a few hundred milliseconds.^3^^–^^5^ Although the mechanism is not fully understood, FLASH-RT induces a differential response between healthy and tumor tissue, resulting in sparing of healthy tissues^6^ while maintaining tumor control.^7^ The FLASH effect has been observed in the brain of rodents with a proton source^7^ and an electron source.^8^ FLASH-RT has also been reported in the thorax of mice using electrons^9^ and with high-energy photons at the PARTER platform at the Chengdu THz Free Electron Laser Facility.^10^ Some conflicting studies, performed with dose rates near the lower limit for the FLASH effect, include synchrotron micro-beam radiotherapy at 37 to 41  Gy/s,^11^ and in cardiac and splenic irradiations at 35  Gy/s.^12^ These studies that did not induce the FLASH effect suggest more investigation into the required dose rates and variability related to the specific anatomical site irradiated is needed to more fully understand the mechanisms behind the FLASH effect.

Following conventional radiation therapy targeting the thorax, radiation pneumonitis is typically observed 4 to 12 weeks post-irradiation^13^ but can occur at later time points,^14^ whereas fibrosis manifests as scar tissue occurring by 6 months.^2^^,^^15^ Micro-computed tomography (micro-CT) has been used to detect fibrosis and other lung injuries in rodent models,^16^^–^^18^ with no adverse effects observed in the cardio-pulmonary system from the radiation received during imaging sessions.^19^^,^^20^ To measure the respiratory function, imaging during specified respiratory phases is required, specifically during peak inspiration and end-expiration to mimic the breath-hold CT scans taken in clinical practice. Respiratory-gated image acquisition can be triggered using the real-time measured respiratory trace^21^^–^^24^ or retrospectively by sorting the acquired projections based on the recorded respiratory trace^25^^,^^26^ or diaphragm position in the images.^27^^–^^30^ Imaged-based quantitative analysis of the lung structure and lung function at different respiratory phases can then be performed.^31^^–^^33^

Our hypothesis is that respiratory-gated micro-CT images taken at different time points post-RT will enable the detection of radiation pneumonitis and fibrosis in mice exposed to different dose rates and prescribed doses. Recently, Esplen et al.^34^ developed a megavoltage, UHDR-compatible photon source at the FLASH irradiation research station at TRIUMF (FIRST; Vancouver, Canada) to facilitate FLASH radiobiological investigations alongside conventional RT in mice. Using the FIRST platform, we have demonstrated that we can achieve both ultra-high and conventional dose rates in mice and characterized the dose deposition.^35^ In this complementary study, we monitor the irradiated mice^35^ up to 24 weeks post-treatment to observe any radiation-induced lung injuries caused by 10-MV photon FLASH-RT and conventional RT using *in vivo* respiratory-gated micro-CT and histology.

## 2 Methods

### 2.1 Animal Model

All animal work was performed in accordance with the Canadian Council on Animal Care, ARRIVE, and the UBC Animal Care Committee (No. A21-0060). Thirty male C57BL6 mice were purchased (Jackson Laboratory, Sacramento, California, United States) and were aged 8 to 11 weeks at baseline scan and 10 to 12 weeks at radiation treatment. The mice were divided (six per group) into treatment groups: 15-Gy FLASH-RT, 15-Gy conventional RT, 30-Gy FLASH-RT, 30-Gy conventional RT, and unirradiated controls.

### 2.2 Respiratory-Gated Micro-CT

Respiratory-gated micro-CT was performed at baseline, followed by bilateral shaving the thoracic region to aid in positioning for RT. During imaging, the mouse was anesthetized by 5% isoflurane in $O_{2}$ in an induction chamber and then positioned prone on a pneumatic pillow to measure the diaphragm motion as a surrogate for respiration,^23^ with isoflurane at 1.5% to 2% for maintenance. Using a physiological monitoring system (BioVet, m2m Imaging, Cleveland, Ohio, United States), we selected points on the real-time respiratory trace near the end of inhale, which were used to trigger the acquisition of the peak inspiration image, and a second trigger, delayed by ∼350  ms, to acquire the end-expiration image, as described previously.^23^

Micro-CT was performed using an eXplore CT 120 scanner (Trifoil Imaging, Chatsworth, California, United States) operated at 80 kVp, 40 mA, and 16 ms per projection. Acquisition settings were determined using an image quality phantom to ensure adequate resolution and low image noise. Each imaging session took 20 to 25 min to complete a pair of images (end-expiration and peak inspiration) with an entrance dose of 0.07 Gy per image pair. Images were reconstructed by filtered back projection with a 75-micron isotropic voxel size, filtered with an edge-preserving bilateral filter in MATLAB (2018a, MathWorks, Natick, Massachusetts, United States). Images were rescaled into Hounsfield units, with the air in the trachea set to −1000  HU and water set to 0 HU.

### 2.3 Radiation Treatment

All photon RT treatments were performed at TRIUMF (Vancouver, Canada) using the FIRST 10 MV UHDR photon source, with $1\times 1\;{\mathrm{cm}}^{2}$ field size and 8.5 cm source-to-surface distance.^34^^,^^35^ Mice were anesthetized with alfaxalone (40 to 60  mg/kg), dexmedetomidine (0.15  mg/kg), and butorphanol (2  mg/kg), positioned prone inside a 50-mL Falcon tube with multiple 5-mm-diameter holes to ensure adequate airflow, and aligned with the beam entering the thorax laterally from the right. EBT3 Gafchromic film (Ashland Advanced Materials, Bridgewater, New Jersey, United States) was placed on the surface of the Falcon tube to provide an estimate of the dose to each mouse.^35^ The prescribed doses at 1 cm depth were 15 Gy using either the UHDR (90  Gy/s) or conventional dose rate (0.04  Gy/s) and 30 Gy with dose rates of 98  Gy/s and 0.06  Gy/s respectively. Following treatment, mice were given a reversal drug atipamezole (1.5  mg/kg) and subcutaneous fluids and monitored until fully recovered. The unirradiated control mice received the anesthesia and recovery procedures only. Mice were monitored daily post-treatment and weighed on an electronic scale every other day.

### 2.4 Post-Treatment Micro-CT

Micro-CT imaging was scheduled at 2, 4, 6, 9, 12, and 24 weeks post-irradiation for the 15-Gy treatment groups and unirradiated control mice. Due to the higher dose groups not eating well over the first week post-irradiation, and losing mice from the high dose groups at later time points, the imaging time points for the high dose groups were altered to 3, 6, 9, 12, and 18 weeks to ensure the mice had fully recovered from the initial anesthesia and RT session prior to imaging in week 3, and to terminate the study early to avoid losing mice (humane endpoint) before week 24. As the symptoms were not expected to occur before 4 weeks, the discrepancy in timing for the first scans should not affect the outcomes of the study. Images were viewed to identify the presence of any lung injuries (regional reductions in air fraction and scar tissue formation) at each time point.

### 2.5 Post-Mortem Analysis

The mice were euthanized immediately after the final micro-CT session by intraperitoneal injection of ketamine (225  mg/kg) and dexmedetomidine (1.5  mg/kg). The lungs were excised and inflation-fixed at 25 cm $H_{2}O$ in 10% formalin. The lungs were paraffin-embedded and sectioned coronally into 4-m-thick slices. For each mouse, three to five slides were stained with hematoxylin and eosin (H&E) and two slides with Masson’s trichrome. Images were acquired using an Axioplan 2 upright microscope (Zeiss, Oberkochen, Baden-Württemberg, Germany) at 2.5×, 5×, and 10× magnification. The entire slice was observed at low magnification to identify regions of damage, and higher magnification images were obtained of the damaged regions.

### 2.6 Micro-CT Image Analysis

Using MicroView (Trifoil Imaging, Chatsworth, United States) as described previously,^32^^,^^36^ the images were reoriented to align the major airways with the axes of the image, and a region of interest (ROI) was drawn starting 2 mm above the carina covering the entire lung. Using a seeded region-growing algorithm and a threshold of − 160  HU,^37^ we segmented the lung from the surrounding tissue. Using the seeded-region growing algorithm within an ROI, we were able to force the segmentation to only select the desired tissue. If the algorithm failed, the bounding ROI was adjusted to ensure proper segmentation. From the segmentation, we measured the volume and CT number, which were used to calculate the functional residual capacity (FRC) using Eq. (1) and tidal volume ($V_{T}$)^32^ using Eq. (2), where the CT # for air was set to −1000  HU. Histograms of the CT numbers within the lung were plotted for both respiratory phases

$$\mathrm{FRC}\;[\mathrm{mL}]={\text{Volume}}_{\text{expiration}}\times \frac{\mathrm{CT}{\#}_{\text{expiration}}}{\mathrm{CT}{\#}_{\text{air}}}.$$ (1)

$$V_{T}\;[\mathrm{mL}]={\text{Volume}}_{\text{inspiration}}\times \frac{\mathrm{CT}{\#}_{\text{inspiration}}}{\mathrm{CT}{\#}_{\text{air}}}-\mathrm{FRC}.$$ (2)

### 2.7 Statistical Analysis

Statistical analysis was performed in Prism (9.1.2, GraphPad Software, La Jolla, California, United States). Descriptive statistics were performed for all groups at each time point. The body weights were compared using Brown–Forsythe and Welch analysis of variance (ANOVA) with Dunnett’s T3 post hoc test (α=0.05). For those time points where lung injuries were identified in the micro-CT images, one-way ANOVA with Tukey’s post hoc test (α=0.05) was performed on the image-based measurements. Endpoint images for the controls and 15-Gy treatments at 24 weeks were compared with endpoint images for 30-Gy treatments at 18 weeks, assuming the mice were no longer growing at these later time points.

## 3 Results

### 3.1 Radiation Therapy Dose

For the 15 Gy prescribed doses, we achieved 14.57±0.80  Gy with a mean dose rate of 89.79±7.67  Gy/s for the UHDR group and 11.74±0.76  Gy with an average dose rate of 0.036±0.003  Gy/s for the conventional RT. For the 30 Gy prescribed doses, the mean dose was 29.65±1.57  Gy, with a mean dose rate of 97.58±14.12  Gy/s for the UHDR group, and the mean dose was 32.94±0.49  Gy, with a mean dose rate of 0.06±0.005  Gy/s for the conventional RT. All of the mice in each group received similar doses, with small deviations from the group mean. The differences between the FLASH-RT and conventional dose rates occurred because the irradiations took place on different days, and some beam instability was noted. The beam stability was improved following the 15-Gy irradiations, which resulted in closer agreement with the prescribed dose for the two groups exposed to 30 Gy.^35^ The full dosimetric characterization of the beamline, including complete details of the dosimetry in live mice, can be found in Esplen et al.^35^

### 3.2 Survival Analysis

**Fig. 1:**
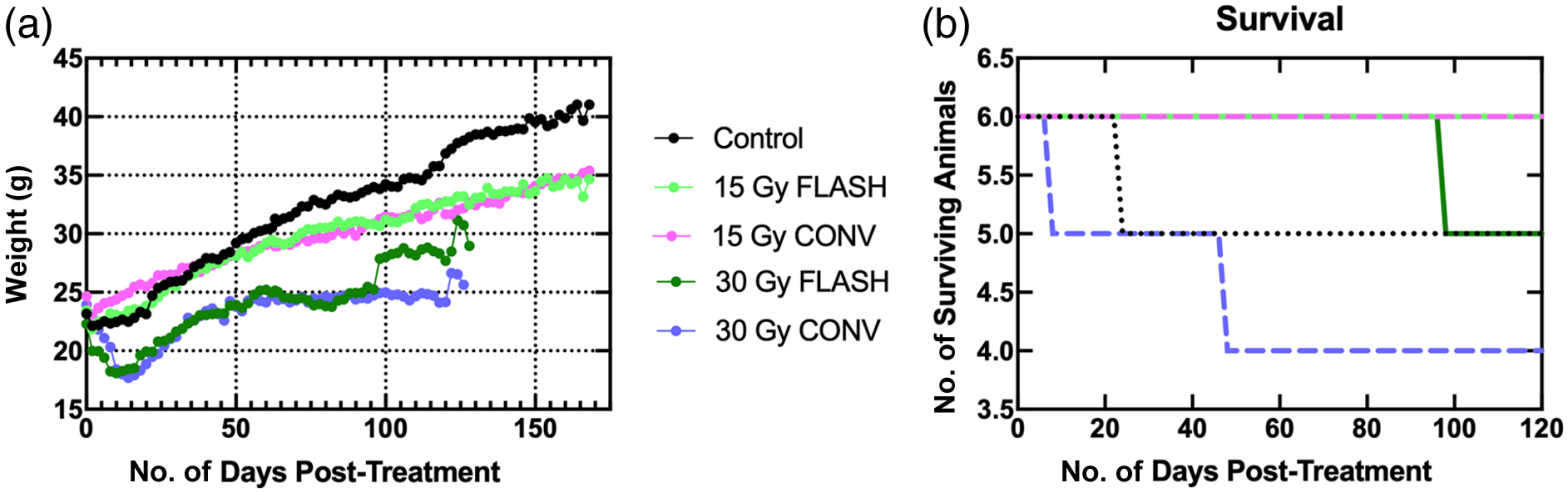
(a) Body weight averaged over the treatment group. The 30-Gy treatment groups had 18 weeks as the endpoint, and the 15-Gy and control groups had 24 weeks as an endpoint. Spikes in the curves correspond to animals lost from the study (96 days for 30-Gy FLASH-RT). (b) Survival curve showing 18 weeks post-treatment. All six animals in the 15-Gy FLASH-RT and 15-Gy conventional treatment groups survived 24 weeks post-treatment.

Following the radiation treatment, mice were weighed every other day; the mice exposed to radiation gained less weight than the controls (p<0.0001 for both 30-Gy groups, p=0.0063 for 15-Gy FLASH-RT, and p=0.0075 for 15-Gy conventional RT). There were no differences in the rate or amount of weight gain between the 15-Gy FLASH-RT and conventional RT (p>0.9999). Both the 30-Gy groups experienced an initial loss of weight lasting for nearly 3 weeks post-irradiation due to radiation gastroenteritis, which was treated with anti-ulcer medication esomeprazole, resulting in reduced weight gain compared with the 15-Gy groups. From the 30-Gy conventional RT, one mouse died 8 days post-irradiation, and a second was euthanized at humane endpoint at 46 days. The 30-Gy conventional RT saw the weight gain plateau around 50 days post-irradiation through the endpoint. The sudden increase at 96 days post-irradiation in the 30-Gy FLASH-RT group corresponded to losing a mouse from the group (humane endpoint)—this mouse was very small in comparison with the others due to the radiation gastroenteritis. All 3 of the irradiated mice that were lost to follow-up were unable to maintain body weight (>30% below pre-RT weight). Despite these differences, there was no significant difference in the weights for the 30-Gy FLASH-RT and conventional RT (p=0.4592). One control mouse also died 25 days into the experimental protocol from a spontaneous tumor that consumed the liver. The weight and survival curves are included in Fig. 1. Note that the survival curve is cropped to 120 days (∼18 weeks) endpoint of the 30-Gy treatment groups, and the curves are unchanged between 18 and 24 weeks for the 15 Gy and control mice.

### 3.3 Quantitative Image Analysis

**Fig. 2:**
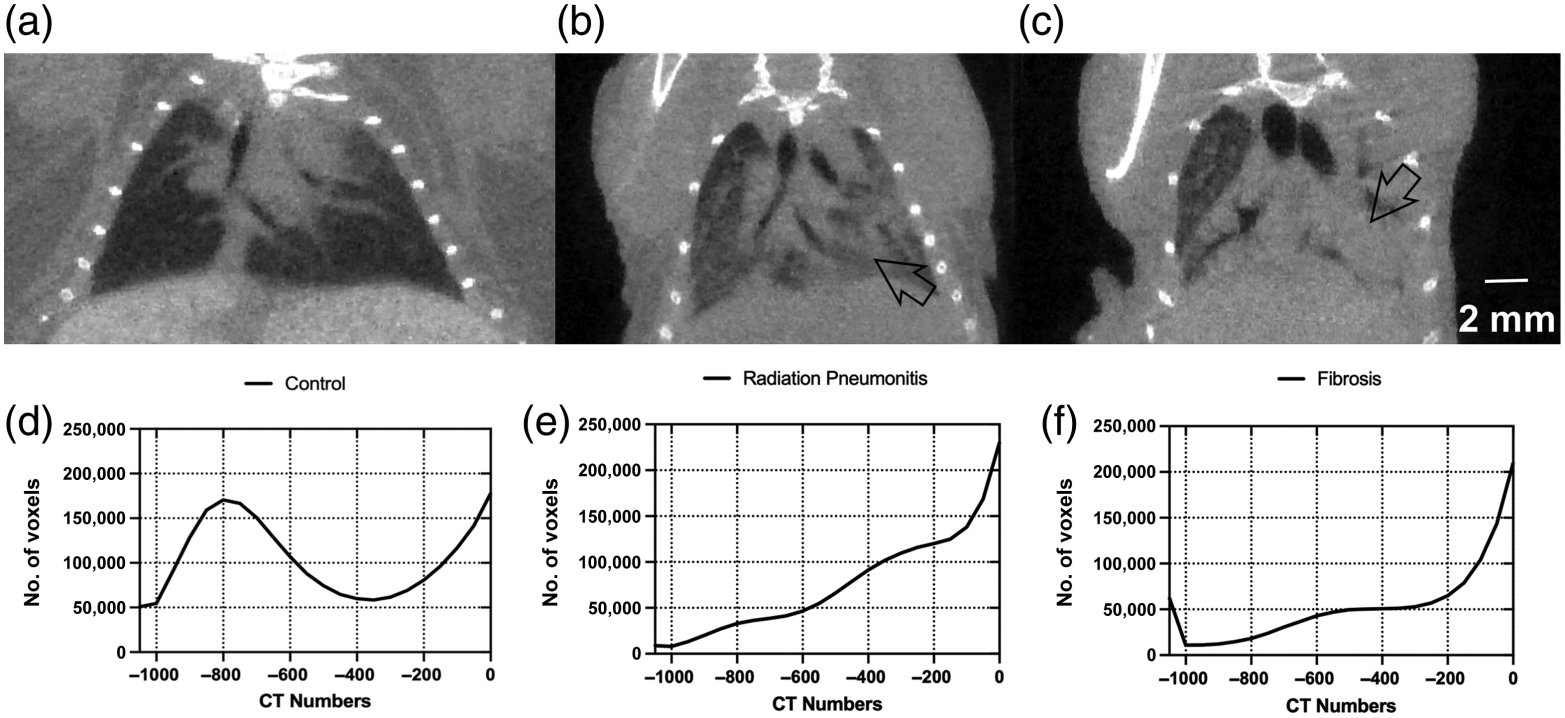
Images of (a) control mouse at 18 weeks with no pathology, (b) 30-Gy FLASH-RT mouse at 12 weeks showing radiation pneumonitis (arrow), and (c) different 30-Gy FLASH-RT mouse at 18 weeks (endpoint) showing fibrosis (arrow). Micro-CT images were obtained during peak inspiration, reconstructed with 0.075-mm voxels, and displayed with the same window and level. The histograms of the CT numbers measured within the lungs during peak inspiration are shown in panels (d)–(f) to demonstrate the shift in CT numbers corresponding to reduced air in the lungs from an accumulation of fluid or scar tissue.

The micro-CT images were viewed to identify radiation-induced lung injuries, specifically radiation pneumonitis, which is inflammation or an accumulation of fluid, and fibrosis, which manifests as a thickening of the tissues (airways, connective tissue, etc.) due to the formation of scar tissue. Both of these pathologies will reduce the volume of air contained within the lungs in the micro-CT images; however, radiation pneumonitis is expected to occur earlier in the time course and typically resolves on its own, allowing us to distinguish among the pathologies. Radiation pneumonitis was observed for one mouse at 9 weeks (15-Gy conventional RT) and two mice at 12 weeks (one from the 15-Gy FLASH-RT and one from 30-Gy FLASH-RT). Figure 2(b) shows an example of radiation pneumonitis, with an overall increase in the intensity (brightness) of the air-filled spaces of the lungs, particularly affecting the lower left lobe in this example. All of the mice that exhibited radiation pneumonitis on one scan had clear lungs at the subsequent time point. Fibrosis was observed as a denser ingrowth of scar tissue that was accompanied by enlarged airways on more severe cases, as seen in Fig. 2(c), and occurred in the endpoint images of two mice (33%) from the 15-Gy FLASH-RT treatment, one mouse (17%) from the 15-Gy conventional RT, four mice (80%) from the 30-Gy FLASH-RT, and three mice (75%) from the 30-Gy conventional RT. Examples of micro-CT images depicting a healthy control mouse, radiation pneumonitis, and fibrosis are shown in Fig. 2 along with histograms of the CT numbers measured within the lung. The histograms show the alteration of the air content in the cases with radiation pneumonitis and fibrosis compared with healthy lungs. A summary of survival and the incidence of pathology found in the micro-CT images and histology are shown in Table 1.

**Table 1:** Incidence of radiation pneumonitis and fibrosis in the micro-CT images, fibrosis detected by histology, and number of animals that survived to the endpoint. The mouse euthanized at 16 weeks from the 30-Gy FLASH group and the mouse euthanized at 6.5 weeks in the 30-Gy conventional group were also included in the histological analysis.

	No. of radiation pneumonitis (micro-CT)	No. of fibrosis (micro-CT)	No. of fibrosis (histology)	No. of survived to endpoint
Unirradiated control	0	0	0	5 of 6
15-Gy FLASH	1 at 12 weeks	1 of 6 (24 weeks)	3 (24 weeks)	6 of 6
15-Gy conventional	1 at 9 weeks	1 of 6 (24 weeks)	1 (24 weeks)	6 of 6
30-Gy FLASH	1 at 12 weeks (euthanized at 16 weeks)	4 of 5 (18 weeks)	4 of 5 (18 weeks) + 1 (euthanized at 16 weeks)	5 of 6
30-Gy conventional	0	1 of 4 (18 weeks)	4 of 4 (18 weeks) + 1 (euthanized at 6.5 weeks)	4 of 6

**Fig. 3:**
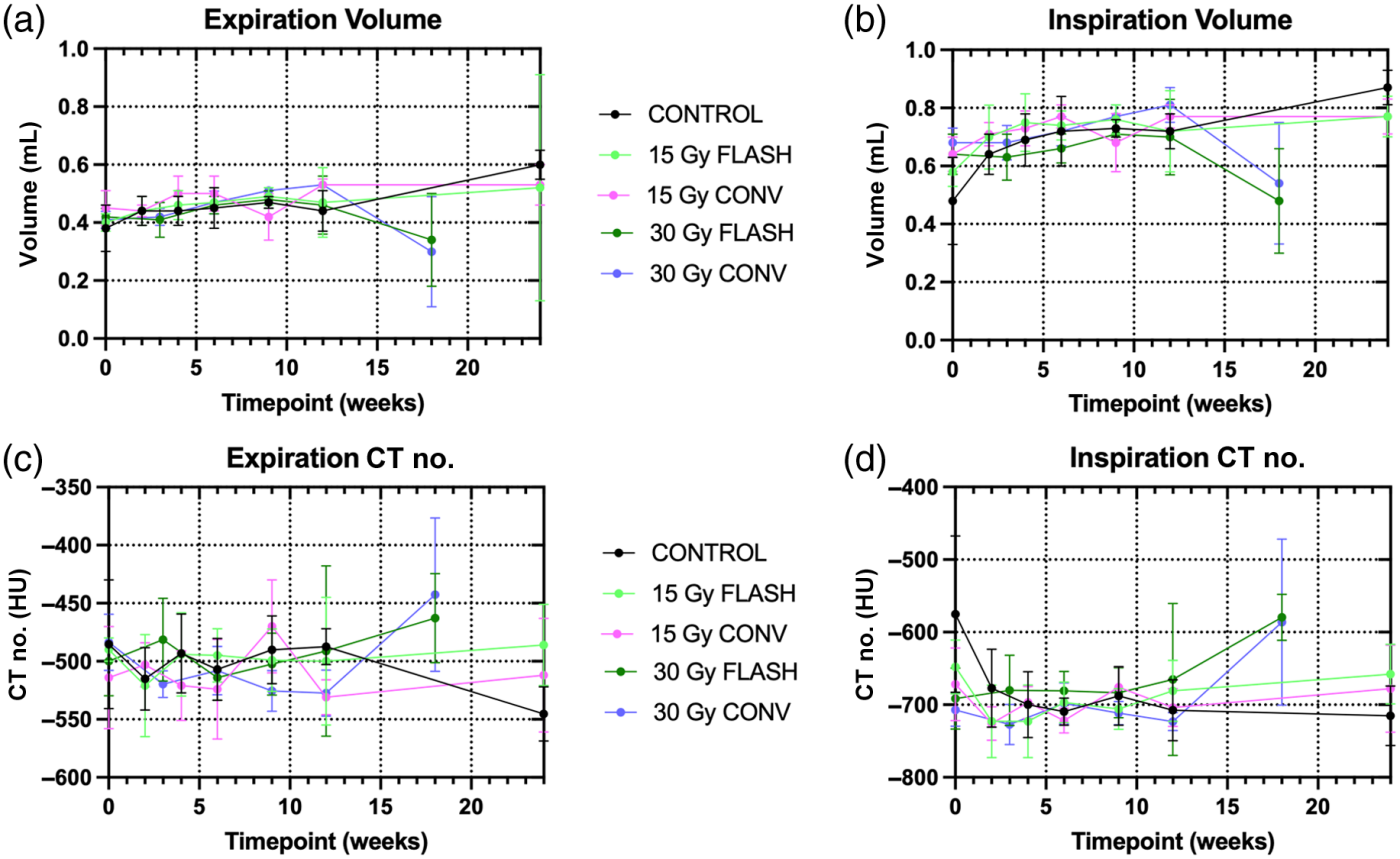
Lung volume during (a) expiration and (b) inspiration and CT numbers during (c) expiration and (d) inspiration measured in the micro-CT images, expressed as mean and standard deviation. Endpoint was 18 weeks post-irradiation for the 30-Gy groups and 24 weeks post-irradiation for the 15-Gy groups. The 30-Gy conventional and 30-Gy FLASH groups had significantly less air in the lungs (p<0.05) at the endpoint compared with the control.

**Fig. 4:**
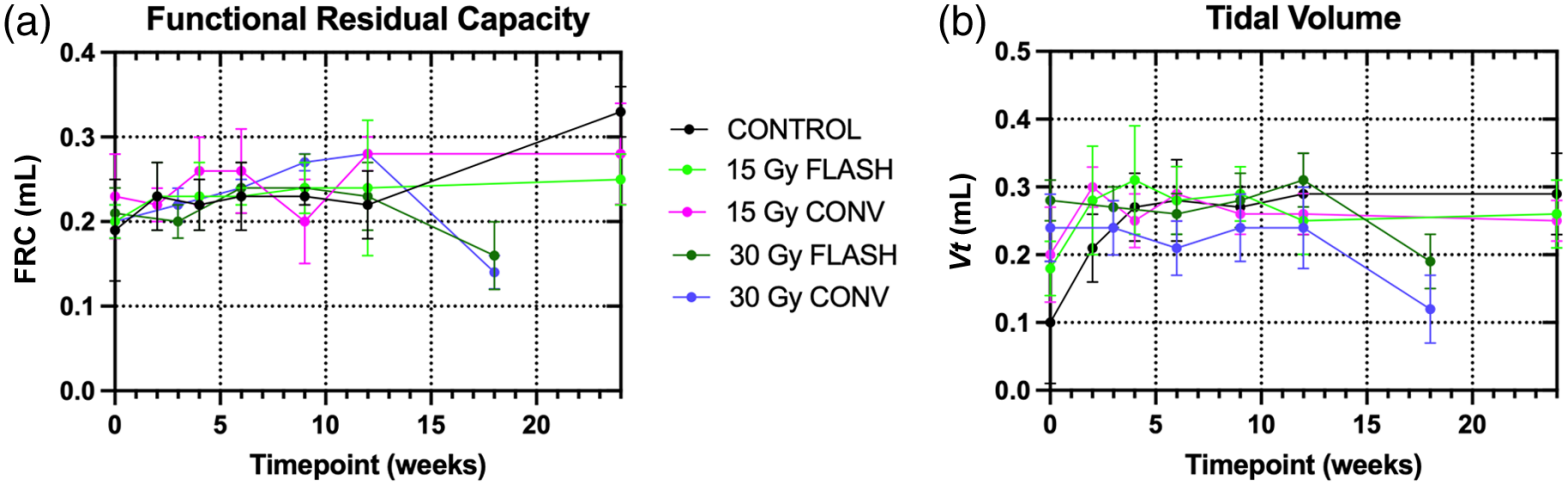
(a) Functional residual capacity and (b) tidal volume calculated from the measured volumes and CT densities, expressed as the mean and standard deviation. Endpoint was 18 weeks post-irradiation for the 30-Gy groups and 24 weeks post-irradiation for the 15-Gy groups. The 30-Gy conventional and 30-Gy FLASH groups had significantly reduced values (p<0.05) at the endpoint compared with the control.

The image-based measurements of the lung volume and air content were averaged over the surviving mice at each time point for each exposure group, shown in Fig. 3 for end-expiration [panels (a) and (c)] and peak inspiration [panels (b) and (d)]. These measured values were used to calculate the lung function metrics, FRC and $V_{T}$, as shown in Fig. 4. Statistics were performed for those time points where pathologies were identified in the micro-CT images (weeks 9 and 12 and endpoint). At 9 weeks, only the end-expiration volume (p =0.0348) and the FRC (p=0.0168) showed significant differences between the 15- and 30-Gy conventional RT due to a mouse with pneumonitis in the 15-Gy conventional RT. For the 12-week time points, too few animals exhibited the pathology to alter the mean. The endpoint images showed significant differences from 30-Gy conventional RT during end-expiration for control (lung volume p=0.0066, CT density p=0.0246, and FRC p=0.0031), 15-Gy conventional RT (lung volume p=0.0294 and FRC p=0.0254), and 15-Gy FLASH-RT (lung volume p=0.0482) and during peak inspiration for control (lung volume p=0.0100 and CT density p=0.0233). For 30-Gy FLASH-RT, significant differences were found during end-expiration with control (lung volume p=0.0148 and FRC p=0.0068) and during peak inspiration for control (lung volume p=0.0010, CT density p=0.0095, and $V_{T}$ p=0.0013), 15-Gy conventional RT (lung volume p=0.0065, CT density p=0.0452, and $V_{T}$ p=0.0017), and 15-Gy FLASH-RT (lung volume p=0.0072 and $V_{T}$ p=0.0088).

No significant differences were found between 30-Gy FLASH-RT and conventional RT for the lung measurements. However, there were marked changes in all measurements between the 12-week and endpoint scans for both 30-Gy groups indicating a reduction in lung volume, air content, and reduced lung function resulting from more severe fibrosis in these two groups. The 30-Gy conventional RT exhibited a 50% reduction in FRC and 39% reduction in $V_{T}$, and the 30-Gy FLASH-RT showed a 30% reduction in FRC and a 50% reduction in $V_{T}$, whereas the 15-Gy groups remained constant for both metrics and the unirradiated controls increased in FRC by 45% and remained constant for $V_{T}$. Given the slightly larger changes in the lung measurements between 12 weeks and endpoint coupled with the reduced survival for the 30-Gy conventional RT, it is possible that these measurements were artificially improved by losing the mice with the most severe pathologies from the group prematurely leaving the only less severe cases for the endpoint scans. The variability is captured in the standard deviations in Fig. 3, with the 12-week and endpoint rows showing increased variation for the 30-Gy treatment groups compared with earlier time points and with the 15-Gy treatment and unirradiated groups. For the lung volume and density at both respiratory phases at the endpoint, the largest variability was exhibited by the 30-Gy conventional RT group.

**Fig. 5:**
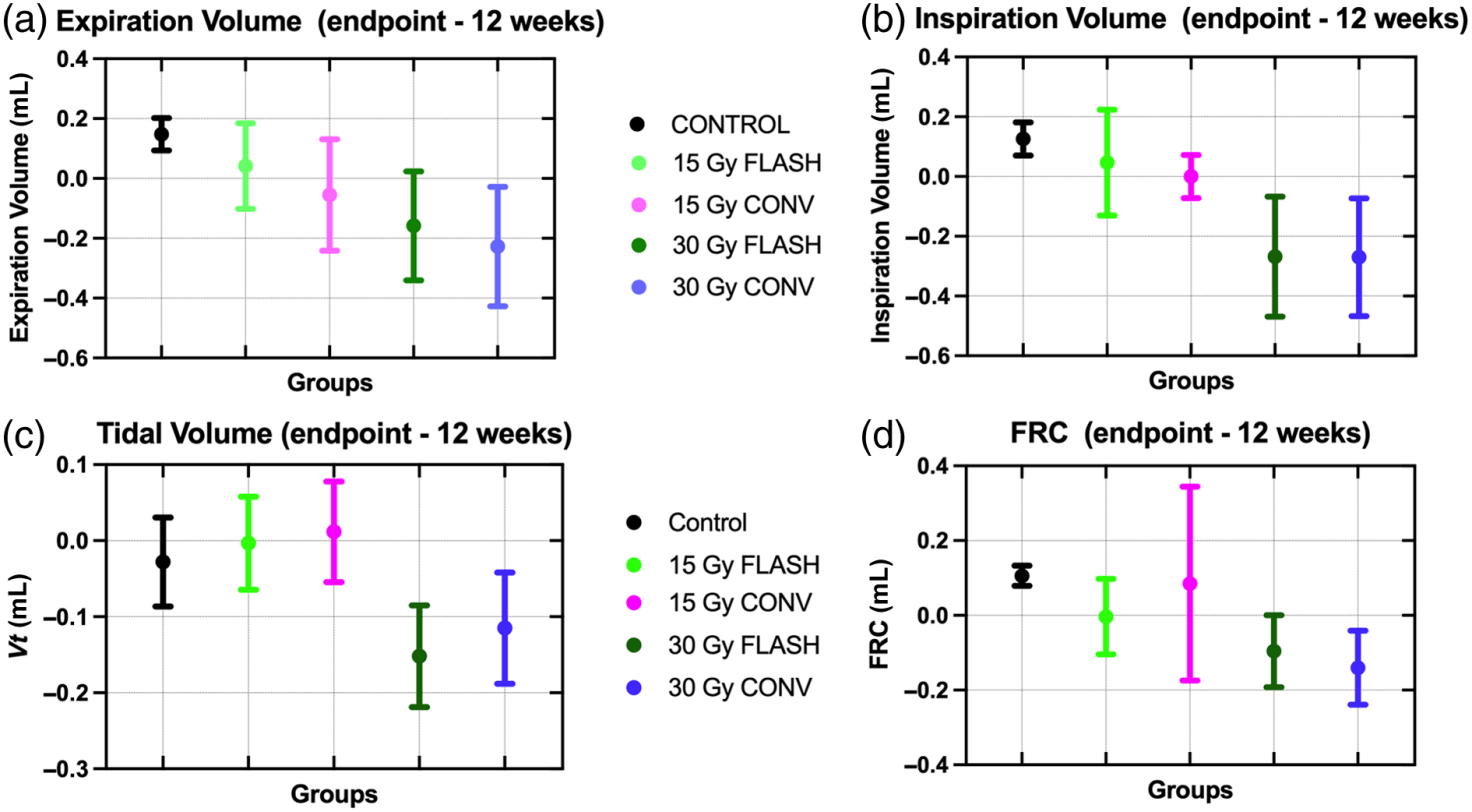
Difference between the measured values at the endpoint and 12 weeks for lung volume at (a) expiration, (b) inspiration, (c) $V_{T}$, and (d) FRC, expressed as the mean and standard deviation. Negative values indicate a reduction in volume at the endpoint compared with 12 weeks.

To uncover additional trends in the data, the change in lung volume, FRC, and $V_{T}$ were calculated for all mice between week 12 and the endpoint. The means and standard deviations are plotted in Fig. 5. No significant differences were seen in FRC. Significant differences from the 30-Gy conventional group were seen for control (end-expiration volume p=0.0165 and peak inspiration volume p=0.0061), 15-Gy conventional ($V_{T}$ p=0.0453), and 15-Gy FLASH (peak inspiration volume p=0.0269). For the 30-Gy FLASH group, significant differences were observed for control (end-expiration volume p=0.0460, peak inspiration volume p=0.0037, and $V_{T}$ p=0.0455), 15-Gy FLASH (peak inspiration volume p=0.0176 and $V_{T}$ p=0.0085), and 15-Gy conventional ($V_{T}$ p=0.0036). The 30-Gy conventional group had the largest reduction in lung volume and was the only group with every mouse exhibiting a volume reduction for all four measured values.

### 3.4 Histology

**Fig. 6:**
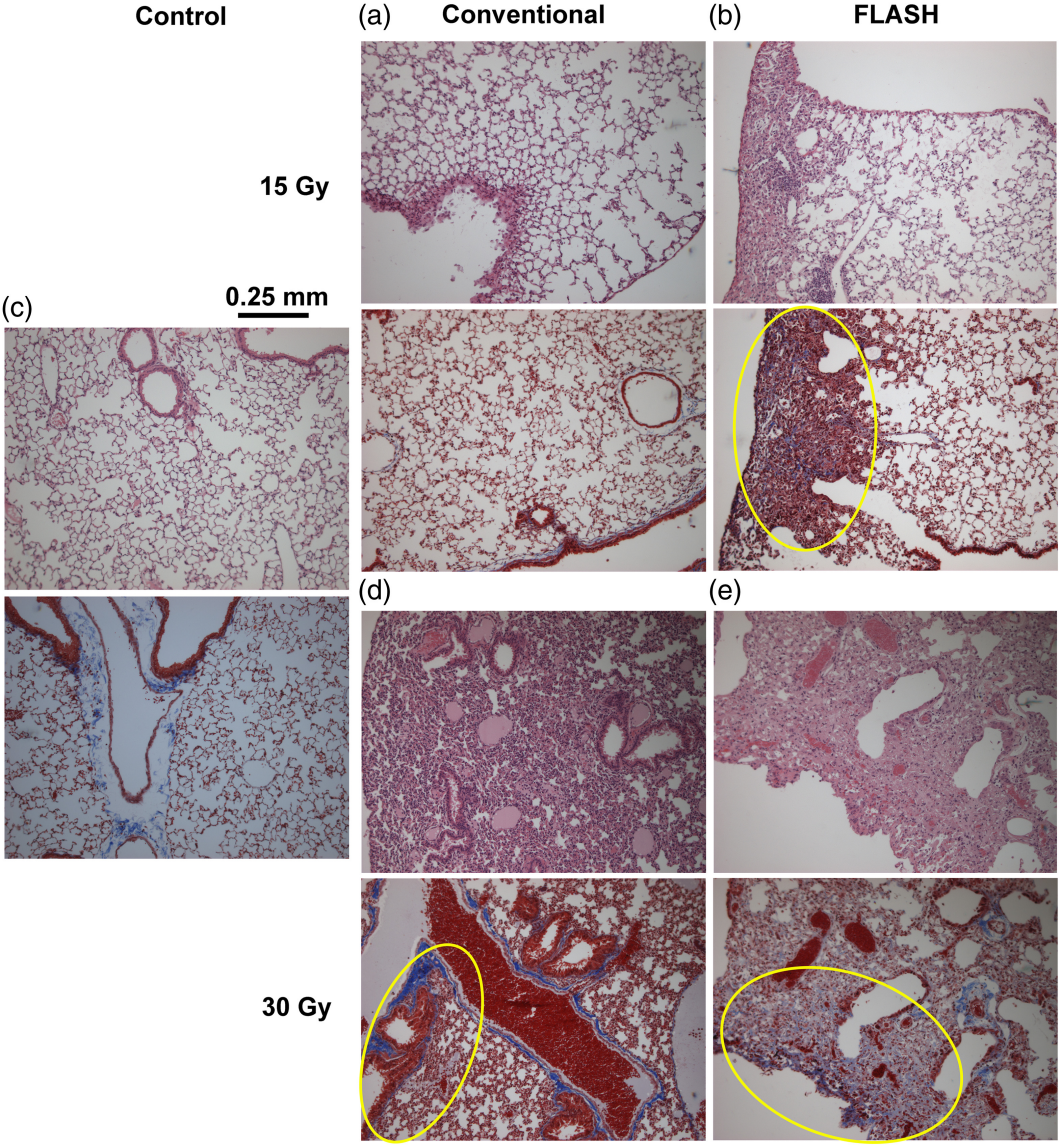
Histology slides (10× magnification) showing lung damage (if present) observed in each treatment group. The increased color density and presence of nuclei (stained purple) indicate fibrotic tissue in the H&E (upper). Collagen (blue) should line the airways and vessels in Masson’s trichrome slides (lower). In the more densely colored pink/red regions, increased collagen (blue) can be seen (circled in yellow). (a) 15-Gy conventional. (b) 15-Gy FLASH-RT. (c) Control. (d) 30-Gy conventional. (e) 30-Gy FLASH-RT.

The H&E slides were used to detect changes in the alveolar structures within the lung. The Masson’s trichrome slides were used to identify collagen (stained blue) indicating fibrotic regions within the parenchyma. Samples of damaged regions from each group are shown in Fig. 6. All control mice exhibited evenly stained slices, with uniformly sized alveolar walls and airspaces. At 15 Gy, the damage was sporadic and localized to small focal regions on the lateral periphery of the right lung (three FLASH-RT and one conventional RT). At 30 Gy, all mice exhibited lung injury in the histology slides (five FLASH-RT and four conventional RT) with damage ranging from small regions along the lateral periphery of the right lung, to wide-spread formation of scar tissue that reduced the airspaces. The lung tissue of the 30-Gy FLASH-RT mouse euthanized at 96 days was also analyzed revealing regional lung damage, and the 30-Gy conventional RT mouse that was euthanized at 46 days had extensive lung damage, which included very dense fibrotic tissue. For the 30-Gy treatment groups, the animals given conventional RT exhibited more extensive, dense fibrosis than FLASH-RT.

## 4 Discussion

Radiation pneumonitis and fibrosis are two important complications after irradiating the thoracic region. Previous studies have shown that radiation pneumonitis, as an acute irradiation response, was detected within 12 weeks post-irradiation, whereas radiation fibrosis was detected at 24 weeks.^13^^,^^38^^,^^39^ Favaudon et al.^9^ found no complications up to 20 Gy for FLASH-RT using an electron beam, whereas fibrosis was observed following conventional RT of 15 Gy. To induce pulmonary side effects with the FLASH-RT dose rates, a higher total dose was needed, with 30 Gy inducing patches of fibrosis at 24 weeks post-exposure.^9^ Jackson et al.^40^ used conventional dose rates with X-rays and gamma rays to deliver 10 to 15 Gy to the thorax and found that the lung damage was highly variable among animals, with occasional fibrosis that was relatively minor. Zhou et al.^41^ showed for conventional RT, a single exposure of 14.55±0.34  Gy was required to achieve 50% fibrosis of the lung at 24 weeks post-irradiation as measured in micro-CT images of female C57BL/6 mice.

Our study is a preliminary investigation to determine if respiratory-gated micro-CT imaging is effective in identifying radiation pneumonitis and fibrosis non-invasively. In contrast to previous studies, we obtained images at two respiratory phases to glean insight into functional metrics of respiratory health. Radiation pneumonitis was observed sporadically as a reduction in the air volume in the lungs at both phases, leading to reduced FRC and $V_{T}$, that resolved by the next imaging time point. We found radiation pneumonitis in the 15-Gy conventional RT group at 9 weeks and in both FLASH-RT groups at 12 weeks, in agreement with Jin et al.’s^13^ timeline of 4 weeks to 12 weeks following 20-Gy conventional RT. We did not detect any radiation pneumonitis for 30-Gy conventional RT, but considering the occurrence is only 13% to 40% in lung cancer treatments^42^^,^^43^ with variable time to develop, it is possible that the pathology was missed with our imaging schedule.

In the endpoint micro-CT scans, fibrosis was observed as an ingrowth of scar tissue into the airspaces, with enlargement of the airways for more severe cases. Compared with histology, the micro-CT scans did not detect some of the fibrosis in the lower dose groups; in these cases, the damage to the lung tissue was limited to a small focal region located at the periphery and may not have been identified due to the small size of the damage or the similarity to normal peripheral tissue. Due to the non-invasive nature of our respiratory-gated micro-CT imaging protocol, we were able to compare the lung volumes, FRC, and $V_{T}$ for different time points. Comparing the endpoint to 12 weeks, we can see a decrease in lung volume for all treatment groups compared with the control. However, the FRC and $V_{T}$ were not affected by these decreases in the 15-Gy treatment groups, suggesting the lung function was preserved for the low-dose treatments. The largest impact on lung function occurred in the 30-Gy conventional RT group, as expected, with every mouse showing a decreased volume of air in the lungs and reduced lung function. Indeed, the fibrosis observed in the 30-Gy conventional RT group was more severe in both the micro-CT and histological images.

One limitation of the study is the inadvertent exposure of the gastrointestinal tract to radiation, which manifested as radiation gastroenteritis in both 30-Gy exposure groups. Future studies will need to improve targeting to avoid sensitive tissues in the esophagus. Although the study suffered from some mortality among the mice exposed to 30 Gy due to radiation gastroenteritis and a spurious liver tumor in the control group, we did see differences in the incidence and the histological markers of fibrosis in the 30-Gy conventional exposure group. A second limitation is the sporadic nature of the radiation pneumonitis. Although we did observe this pathology in the images during weeks 9 and 12, it is possible that some mice developed the pathology among scanning time points, and so, it was missed on our imaging schedule. More frequent imaging time points between 8 and 12 weeks may be beneficial and could replace the earlier time points (2 to 6 weeks).

In spite of the challenges associated with the novelty of our irradiations (first live animals on the FIRST platform), our results show respiratory-gated micro-CT images were able to detect both radiation pneumonitis and fibrosis and suggest further study is warranted with larger cohorts to better identify the differences among dose rates. Future studies will also include tumor-bearing animals alongside healthy animals to assess the tumor control for each exposure group.

## 5 Conclusions

We performed *in vivo* FLASH-RT and conventional RT in mice using the 10-MV photon FIRST platform and used respiratory-gated micro-CT to identify radiation pneumonitis and fibrosis and quantify the severity of radiation-induced lung injury. Significant changes were observed in the functional lung metrics for 30-Gy treatment groups compared with control for FRC (50% reduction for FLASH-RT and 56% reduction for conventional RT) and $V_{T}$ (47% reduction for FLASH-RT). The micro-CT analysis correlated well with observations on histology, with reductions in the lung volume, FRC, and $V_{T}$ suggesting more fibrosis has developed and reduced the airspace within the lung parenchyma. Future studies will include respiratory-gated imaging to monitor tumor control in addition to normal tissue effects in a rodent tumor model.

## Data Availability

The raw datasets used in this study are very large 3D images and therefore are impractical to submit as supplemental material. The datasets used and/or analyzed during the current study are available from the corresponding author upon reasonable request.
